# Co-occurring climate events and environmental justice in California, 2018–2019

**DOI:** 10.1088/2752-5309/ada96f

**Published:** 2025-02-04

**Authors:** Brittany Shea, Gabriella Y Meltzer, Benjamin B Steiger, Robbie M Parks, Vivian Do, Heather McBrien, Nina Flores, Milo Gordon, Elizabeth M Blake, Joan A Casey

**Affiliations:** 1 Department of Environmental Health Sciences, Columbia University Mailman School of Public Health, New York, NY, United States of America; 2 The Collaborative for Women’s Environmental Health, Department of Obstetrics and Gynecology, Columbia University Irving Medical Center, New York, NY, United States of America; 3 Department of Environmental and Occupational Health, University of Washington School of Public Health, Seattle, WA, United States of America; 4 Department of Epidemiology, University of Washington School of Public Health, Seattle, WA, United States of America

**Keywords:** climate change, disasters, social vulnerability, temperature, power outages, wildfires

## Abstract

Climate change will increase the frequency of extreme weather events. This means climate-driven events like wildfires and power outages will likely co-occur more often, potentially magnifying their health risks. We characterized three types of climate-driven events—anomalously warm temperatures, wildfire burn zone disasters, and long power outages—in 58 California counties during 2018–2019. We defined county-day anomalously warm temperatures when daily average temperatures exceeded 24 °C and the 85th percentile of the long-term county average. We defined county-day wildfire burn zone disasters when an active wildfire burn zone intersected a county, burned 1+ structures, killed a civilian, or received a Federal Emergency Management Agency Fire Management Declaration, and overlapped with a community. For a subset of the 38 counties (66%), long power outage county days were identified using PowerOutage.us data when an outage affected >0.5% of county customers for 8+ h. Co-occurring events were when 2+ of these events occurred on the same county day. Using the CDC/ATSDR Social Vulnerability Index (SVI), we determined whether co-occurring events disproportionately affected vulnerable populations. Nearly every county (97%) experienced at least one day of anomalously warm temperatures, 57% had at least one wildfire burn zone disaster day, and 63% (24/38 counties with available data) had at least one long power outage day. The most common co-occurring events (anomalously warm temperatures and wildfire burn zone disasters) impacted 24 (41%) counties for 144 total county-days. We did not find a clear connection between co-occurring events and social vulnerability. We observed an inverse correlation between co-occurring wildfire burn zone disasters and long power outage days with SVI, and a positive correlation between co-occurring anomalously warm and long power outage days with SVI. This analysis can inform regional resource allocation and other state-wide planning and policy objectives to reduce the adverse effects of climate-driven events.

## 1. Background

Warm temperatures, wildfires, and power outages have increased in frequency, severity, and geographic range due to climate-change-induced rising temperatures and prolonged drought conditions [1–5]. Of all US states, California has been most affected by wildfires: 76% of structures destroyed by wildfires and 20% of the total area burned by wildfires from 1990 to 2020 occurred in California [6, 7]. Extreme heat is increasingly affecting the health of California residents [8]. In addition, the number of weather-related power outages is rising across the US, and from 2000 to 2023 California had the third most (*n* = 145) weather-related power outages of any state [9, 10]. Furthermore, these events disproportionately impact persistently marginalized communities, including those of color and low-income [11].

Co-occurring wildfires, power outages, and heat pose serious health risks due to the potential inaccessibility/inoperability of cooling mechanisms and air filters powered by electricity. Anomalous heat [12, 13], wildfire burn zones [14, 15], and power outages [4, 16, 17] individually harm health, and co-occurring events may have synergistic negative effects on health [18]. Despite these potential risks, no studies have characterized these three potentially co-occurring events. Evaluating the frequency and distribution of climate-driven events can inform interventions to protect against their adverse health effects.

Considering differences in co-occurrence among vulnerable populations is imperative to inform interventions and target resources effectively [11, 19]. Vulnerable individuals experiencing a climate-driven event may experience a higher exposure or have a worse response. Wildfires have broad public health consequences, and vulnerable populations are particularly at risk of experiencing negative health outcomes [20–22]. For example, older adults, young children, and pregnant persons are more at risk due to physiological differences, and individuals of low socioeconomic status are more at risk due to fewer assets to adapt to wildfires when they occur. These same groups are also more vulnerable to heat’s adverse health impacts, as are people of color, due to disproportionate exposure and less access to resources resulting from historical racism [23, 24]. Finally, communities that are more socioeconomically vulnerable may experience longer power outages, and may also have a stronger response due to worse housing quality or underlying conditions [4, 25, 26].

Here, we evaluate three interconnected climate-driven events: anomalously warm temperatures, wildfire burn zone disasters, and long power outages (8+ h). First, we identify California counties exposed to one, two, or three climate-driven events on the same day during 2018–2019. Next, we compare the social vulnerability of counties exposed to varying levels of the three climate-driven events.

## 2. Methods

During 2018–2019 in California, we examined three county-daily-level climate-driven events: anomalously warm temperatures, wildfire burn zone disasters, and long power outages. We also assessed whether exposure to these events differed by level of social vulnerability. We focused on the years 2018–2019 because these were the years for which data were available.

A county day was defined as anomalously warm if the daily mean ambient temperature on that given day exceeded the 85th percentile of the weekly long-term county average for the day (1981–2010) [27,28], and exceeded an absolute threshold of 24 °C. Since we are capturing anomalous temperatures that have health relevance and are an interplay between absolute and relative thresholds, we used ⩾85th percentile to have a percentile relative extreme, and this measure has largely behavioral implications [5]. Of the days that were ≥85th percentile, the median temperature was 17.3 °C (IQR: 12.7, 23.6). We added a double condition with an absolute cutoff of >24 °C because we wanted to have an absolute extreme that ensured we captured truly warm temperatures as the study covers the entire year. Absolute temperatures have largely physiological implications and there are limits of adaptation physiologically [12].

We identified wildfire burn zone disasters using data from the California Department of Forestry and Fire Protection, and the Federal Emergency Management Agency (FEMA). Wildfire burn zone disasters were those that either burned 1+ structures, killed a civilian, or received a FEMA Fire Management Declaration, and that overlapped with a community (population density of ≥250 people km^−2^) [29, 30]. We defined a county-day as exposed to a wildfire burn zone disaster when a wildfire was burning (i.e. the day fell between ignition and containment date), and the wildfire burn zone boundary overlapped spatially with the county perimeter.

To identify county-days with long power outages, we used PowerOutage.us data and identified outages that lasted for 8+ h while affecting >0.5% of county customers, as in prior work [4, 31]. Reliable power outage data was available for 38/58 (65.5%) of counties, and all events involving power outage data are only defined for this subset of counties due to limited data availability. Power outage data was considered reliable if county utility providers’ application programming interfaces report ⩾50% of the time and if reported customers covered ⩾50% of total county customers [4].

We defined climate-driven events as co-occurring when they took place in the same county on the same day. We counted the total number of single and co-occurring climate-driven events on each county-day from 2018 to 2019.

We defined county-level social vulnerability using the California-specific 2018 Centers for Disease Control and Prevention/Agency for Toxic Substances and Disease Registry Social Vulnerability Index (SVI), which used American Community Survey 2014–2018 data for county estimates of: individuals living below the poverty line, unemployment, income, individuals without a high school diploma, persons aged 65 or older, persons aged 17 or younger, civilians with a disability, single-parent households, minority status, persons who speak English ‘less than well,’ households in multi-unit housing, mobile homes, crowded households, households with no vehicle available, and persons in group quarters [32]. The SVI assigns a number between 0 (least vulnerable) and 1 (most vulnerable) to each county to compare relative social vulnerability concerning community preparedness for and recovery from disasters [32]. We plotted the relationship between the total number of climate-driven events and SVI and estimated the Spearman correlation coefficient among affected counties.

## 3. Results

**Figure 1.**
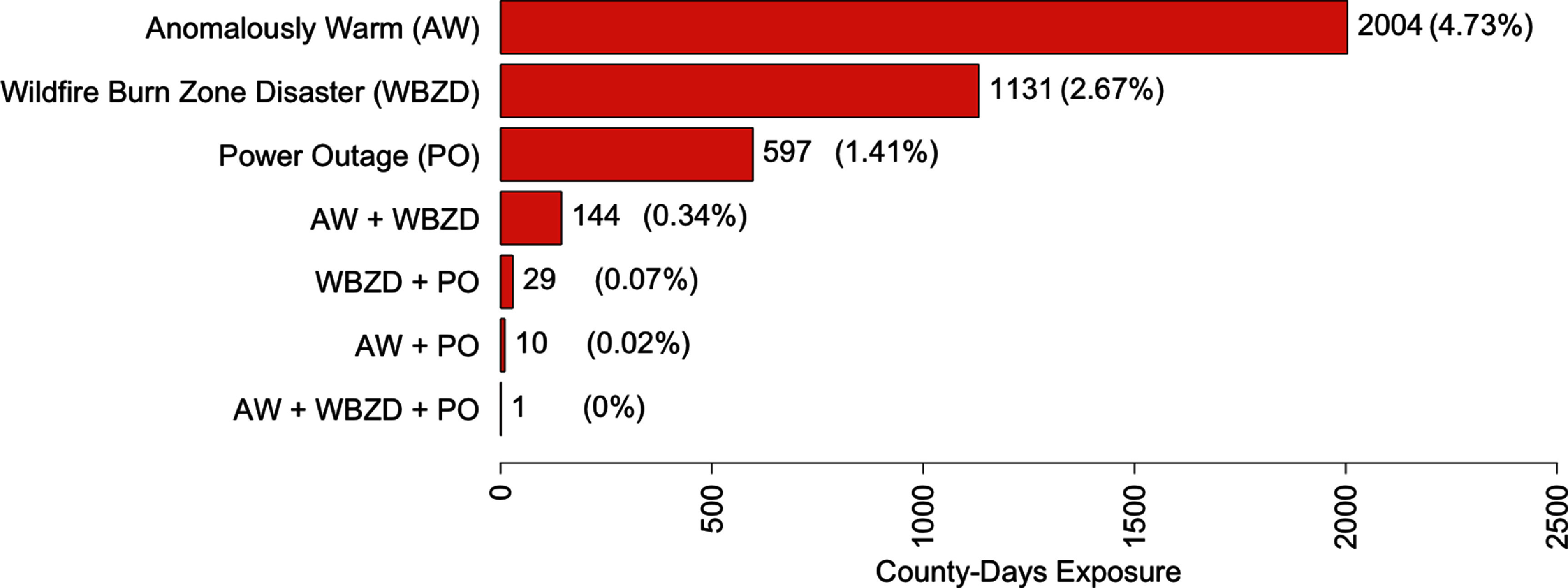
County-days of exposure to climate-driven events in California, 2018–2019. Percentages calculated out of total county-days (*n* = 42,340) from 2018 to 2019. AW, anomalously warm; PO, power outage; WBZD, wildfire burn zone disaster. Events are not mutually exclusive.

This analysis found that 56/58 California counties (97%) experienced at least one anomalously warm day in 2018–2019, totaling 2004 county-days or 4.73% of all county-days (figure 1). Wildfire burn zone disasters were also prevalent, experienced by 57% (*n* = 33) of counties on 1131 county-days. Long power outages lasting 8+ h affected 63% (24/38 counties with available data) of counties for a total of 597 county-days. SVI values ranged from 0 (no vulnerability, Sierra County) to 1 (highest vulnerability, Imperial County), with a median of 0.5.

The most common co-occurring event was anomalously warm temperature and wildfire burn zone disaster days, experienced by 24 (41%) California counties for a total of 144 county-days. 13.2% (5/38 counties with available data) of counties experienced co-occurring anomalously warm temperature and long power outage days (29 county-days in total), and 18.4% (7/38 counties with available data) counties experienced co-occurring wildfire burn zone disaster and long power outage days (10 county-days in total) during the study period. Sometimes, a county experienced more than one wildfire burn zone disaster on the same day; on 63 d, two different wildfire burn zone disasters occurred in the same county (including in Los Angeles, Riverside, Shasta, and Ventura counties).

**Figure 2.**
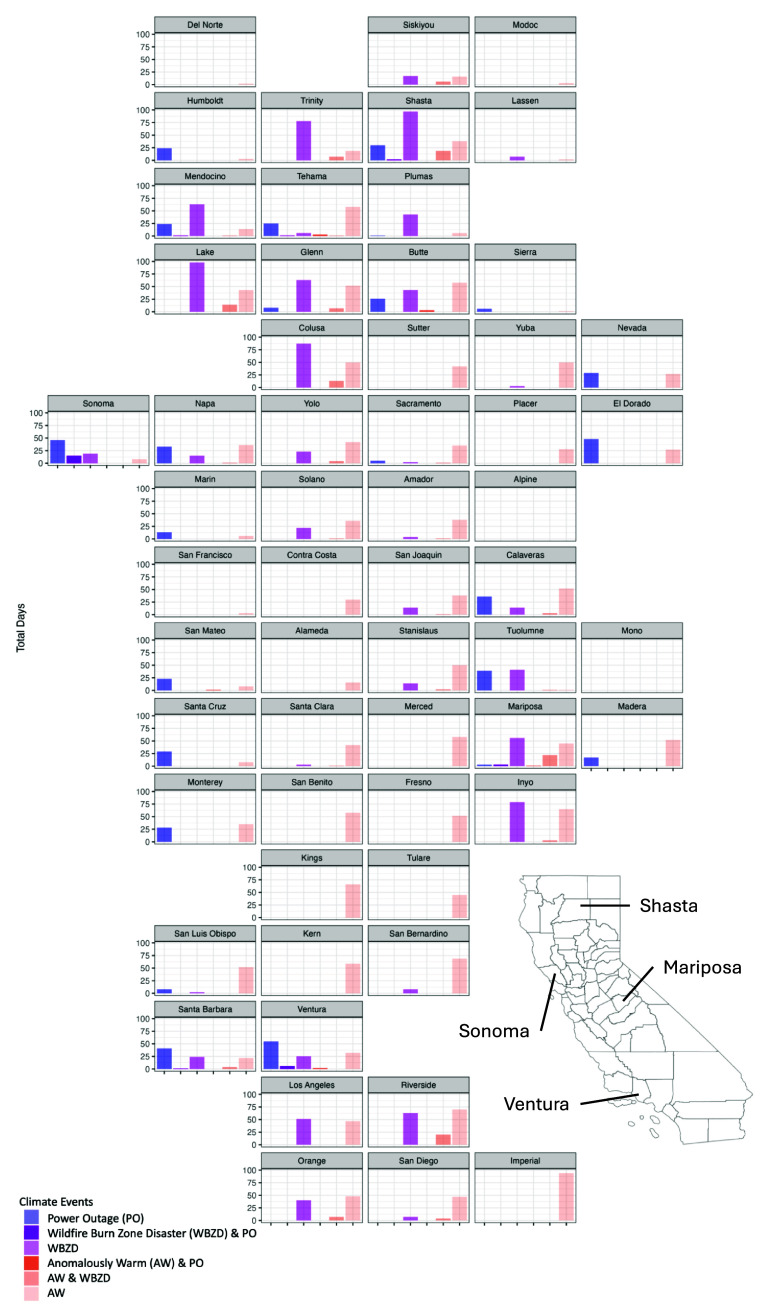
Distribution of daily co-occurring climate-driven events by California county, 2018–2019. AW, anomalously warm; PO, power outage; WBZD, wildfire burn zone disaster.

When examining the distribution of total co-occurring events and the variety of co-occurring event types, four counties exhibited the highest counts in both categories: Mariposa, Shasta, Sonoma, and Ventura Counties (figure 2). Only Mariposa County experienced all three climate-driven events on the same day (10 August 2018, during the Ferguson Fire). Mariposa County also had the greatest number of co-occurring events, with 22 co-occurring anomalously warm temperature and wildfire burn zone disaster days during the study period (supplementary table 1).

Among the top quarter most vulnerable counties in California (SVI > .75), there were 4 county-days with co-occurring anomalously warm temperatures and wildfire burn zone disasters, 3 county-days with co-occurring anomalously warm temperatures and long power outages, and 1 county-day with co-occurring wildfire burn zone disasters and long power outages. Conversely, among the bottom quarter of least vulnerable counties, there was 1 county-day with all three events (anomalously warm temperature, wildfire burn zone disaster, and long power outage), in addition to 27 county-days with co-occurring anomalously warm temperatures and wildfire burn zone disasters, 2 county-days with co-occurring anomalously warm temperatures and long power outages, and 18 county-days with co-occurring wildfire burn zone disasters and long power outages (supplementary table 2). Among counties lacking power outage data, the average SVI was 0.573.

**Figure 3.**
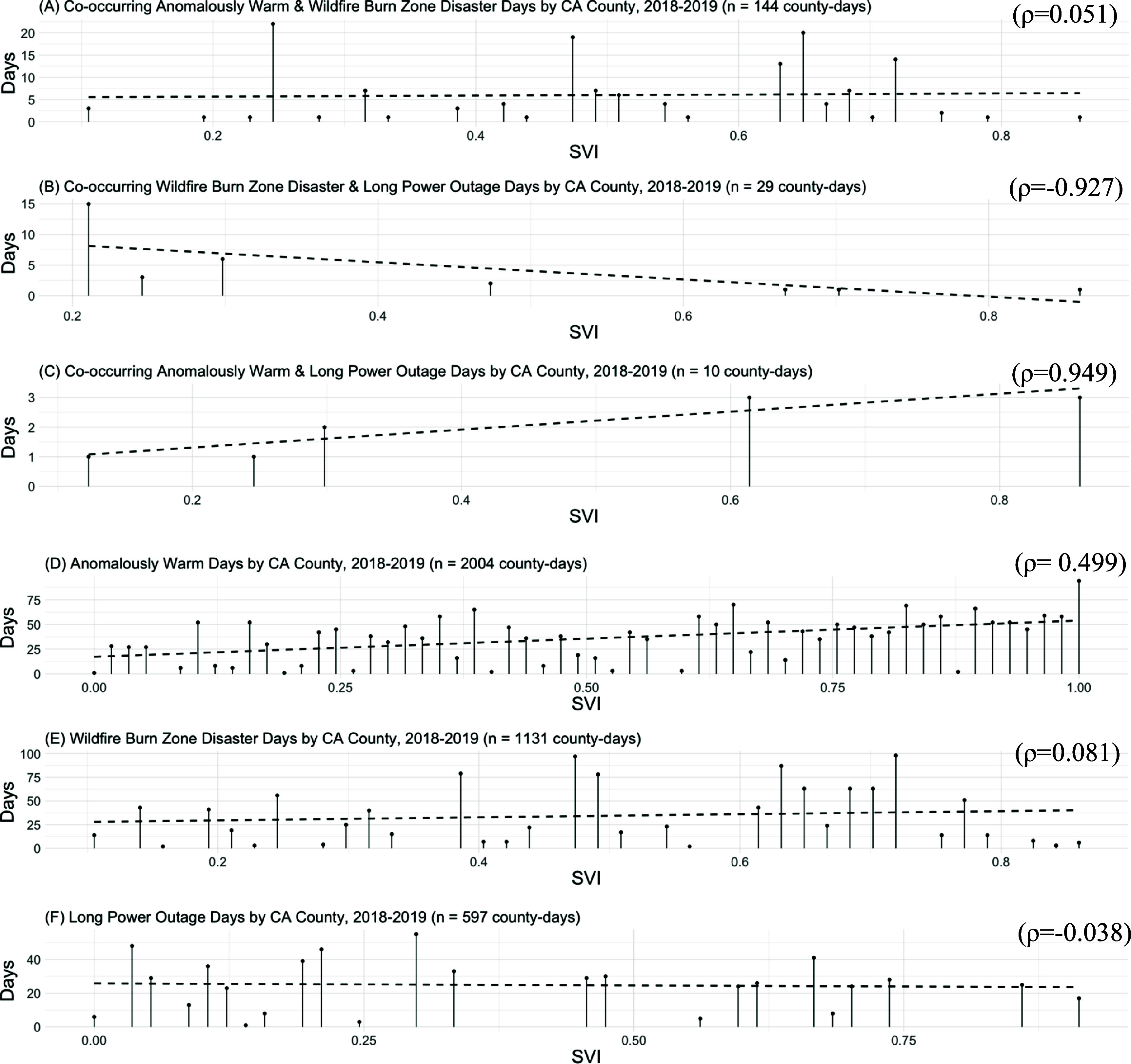
Climate-driven events and the Social Vulnerability Index (SVI). The *x*-axis is county SVI. The *y*-axis is number of days. The dots are counties. The dashed line represents the best linear fit. Panels are labeled with Spearman *ρ*.

We plotted the relationship between county-level number of co-occurring and individual disaster days and SVI (figure 3). Among the 24 counties experiencing co-occurring anomalously warm and wildfire burn zone disaster days, we observed a very weak positive correlation between the number of co-occurring disaster days with SVI (Spearman *ρ* = 0.051), indicating little to no relationship between the two variables. For co-occurring wildfire burn zone disasters and long power outage days, we observed a strong inverse correlation with SVI (Spearman *ρ* = −0.927), suggesting that a greater number of co-occurring wildfire burn zone disasters and long power outage days were associated with less county-level vulnerability. For co-occurring anomalously warm and long power outage days, we observed a strong positive correlation with SVI (Spearman *ρ* = 0.949), suggesting that a greater number of co-occurring anomalously warm and long power outage days were associated with more county-level vulnerability. These findings are only suggestive since our sample size was very small, with 7 counties (29 county-days) and 5 counties (10 county-days), respectively. We also observed a positive correlation between anomalously warm days and county SVI (Spearman *ρ* = 0.499), meaning that more anomalously warm days were associated with higher county-level vulnerability. We did not see a similar relationship between other individual events and SVI (figure 3).

## 4. Discussion

In this analysis, we examined single and co-occurring climate-driven events at the county-level from 2018 to 2019 in California. We found nearly every (97%) California county experienced anomalously warm temperatures, with 2004 county-days total over the two-year period. The most common co-occurring events were anomalously warm temperatures and wildfire burn zone disasters, experienced by 41% of counties on 0.3% of study county-days. We observed an inverse correlation between co-occurring wildfire burn zone disasters and long power outage days and SVI, a positive correlation between co-occurring anomalously warm and long power outage days and SVI, and a positive correlation between anomalously warm days and SVI. We did not observe a relationship between other co-occurring or individual events and SVI.

Prior studies have examined co-occurring events and issues related to environmental justice in California. Masri *et al* evaluated warm temperatures and high fine particulate matter concentration compound risk days in California from 2018 to 2020 and found census tracts with more compound risk days were more socially disadvantaged using the American Community Survey five-year averaged data from 2018 [19]. Rosenthal *et al* looked at the co-occurrence of extreme heat and wildfire smoke in California in 2020 and found that 68% of the state experienced days with both extreme heat and extreme smoke at some point during the year [33]. We did not observe a correlation between co-occurring anomalously warm and wildfire burn zone disaster days with SVI, but we did find that counties with more anomalously warm days alone tended to have higher SVI scores. These differences may have arisen because our study was conducted at a different spatial scale than prior studies (e.g. counties versus census tracts) and because we assessed co-occurring anomalously warm and wildfire burn zone disaster days, rather than wildfire smoke days. Since air pollution from wildfire smoke can travel long distances, a greater number of counties would have had wildfire smoke days than had wildfire burn zone disaster days [2].

We identified 184 county-days with some type of co-occurring climate-driven events in California between 2018 and 2019. Single events threaten health, but co-occurrences can have multiplicative effects on adverse health outcomes. Chen *et al* examined compound extreme heat and wildfire smoke days and cardiorespiratory hospitalizations in California from 2006 to 2019 and observed a greater risk of hospitalizations from exposure to both hazards simultaneously compared to exposure to single hazards. Health effects of compound hazards were stronger among more socially disadvantaged communities [18].

Climate-driven events in communities that are more socially vulnerable are at greater risk of experiencing negative health outcomes. This study found that disadvantaged areas experienced more co-occurring anomalously warm and long power outage days, suggesting these communities could have less resilient power systems that cannot withstand the pressures of extreme heat. We also found that anomalously warm days occurred more often in areas with higher social vulnerability where individuals are more at risk of suffering from heat. For example, individuals of low socioeconomic status could live in crowded households or multi-unit housing that trap heat or lack air conditioning. Given the prevalence and potential health impacts, public health risk communication is required surrounding co-occurring exposures. Coker *et al* surveyed public health agency communications from 2013 to 2023 in Canada and the United States on co-exposure to wildfire smoke and extreme heat and found that out of 15 online resources, only 2 were from California [34].

Our study had limitations. It was conducted at the county-level, possibly missing within-county heterogeneity in the three climate-driven events. Further, reliable power outage data was only available from 2018 to 2019 for 38/58 (65.5%) California counties. Therefore, we likely underestimated the number of co-occurring events. Our analysis did not consider wildfire smoke exposure; therefore, our analysis also underestimates the number of people impacted by wildfires, an area for potential future research.

Our findings regarding co-occurring climate-driven events direct attention to which counties recently experienced such events in California. The findings can inform regional resource allocation and other state-wide planning and policy objectives to reduce adverse effects.

## Data Availability

The data that support the findings of this study are openly available at the following URL/DOI: https://github.com/brittshea/ca_compound_climate [35].
